# Supplementary material for: Decrease in Mycophenolate Mofetil Plasma Concentration in the Presence of Antibiotics: A Case Report in a Cystic Fibrosis Patient with Lung Transplant
Source: Int J Mol Sci. 2024 Feb 17;25(4):2358. doi: 10.3390/ijms25042358 (PMC10888672; doi:10.3390/ijms25042358)
Supplement: Supplementary file 1 [file ijms-25-02358-s001.zip › ijms-2860739-supplementary.pdf]

Day	MP	Everolimus	sCr	AST/GOT	ALT/GPT	GGT	CRP	WBC	Neutrophils	Lymphocytes	Monocytes
**Range**	**≥ 1.3mg/L**	**3.0-8.0 μg/L**	**0.5-1.3 mg/dL**	**<45 U/L**	**<45 U/L**	**10-55 U/L**	**<5.0 mg/L**	**4-11 x10^3/μL**	**2-7.5 x10^3/μL**	**1-4 x10^3/μL**	**0.1-1 x10^3/μL**
-88	1.16 mg/L	3.78 μg/L	1.26 mg/dL	16.00 U/L	19.00 U/L	11.00 U/L	52.50 mg/L	8.12 x10^3/μL	4.92 x10^3/μL	2.50 x10^3/μL	0.60 x10^3/μL
-83	-	1.88 μg/L	1.31 mg/dL	42.00 U/L	50.00 U/L	12.00 U/L	15.00 mg/L	8.93 x10^3/μL	4.59 x10^3/μL	3.45 x10^3/μL	0.77 x10^3/μL
-81	-	2.89 μg/L	1.24 mg/dL	40.00 U/L	53.00 U/L	11.00 U/L	10.20 mg/L	7.08 x10^3/μL	3.92 x10^3/μL	2.43 x10^3/μL	0.63 x10^3/μL
-68	2.30 mg/L	2.06 μg/L	1.04 mg/dL	51.00 U/L	86.00 U/L	17.00 U/L	2.20 mg/L	6.28 x10^3/μL	4.31 x10^3/μL	1.46 x10^3/μL	0.47 x10^3/μL
-20	2.90 mg/L	2.66 μg/L	1.07 mg/dL	43.00 U/L	35.00 U/L	13.00 U/L	1.70 mg/L	5.93 x10^3/μL	3.49 x10^3/μL	1.83 x10^3/μL	0.50 x10^3/μL
3	2.94 mg/L	6.27 μg/L	1.41 mg/dL	25.00 U/L	13.00 U/L	13.00 U/L	356.50 mg/L	7.35 x10^3/μL	5.19 x10^3/μL	1.46 x10^3/μL	0.68 x10^3/μL
4	-	-	1.40 mg/dL	-	-	-	344.50 mg/L	6.07 x10^3/μL	4.53 x10^3/μL	1.01 x10^3/μL	0.49 x10^3/μL
5	-	-	1.30 mg/dL	-	-	-	381.90 mg/L	-	-	-	-
9	0.54 mg/L	-	1.60 mg/dL	-	-	-	172.50 mg/L	5.06 x10^3/μL	3.11 x10^3/μL	1.40 x10^3/μL	0.41 x10^3/μL
11	-	-	1.63 mg/dL	39.00 U/L	46.00 U/L	44.00 U/L	111.80 mg/L	-	-	-	-
12	0.25 mg/L	-	1.42 mg/dL	-	-	-	96.50 mg/L	5.45 x10^3/μL	2.62 x10^3/μL	2.35 x10^3/μL	0.31 x10^3/μL
14	-	-	1.12 mg/dL	29.00 U/L	35.00 U/L	30.00 U/L	56.70 mg/L	7.21 x10^3/μL	4.24 x10^3/μL	2.10 x10^3/μL	0.70 x10^3/μL
17	-	-	1.11 mg/dL	-	-	-	27.40 mg/L	-	-	-	-
19	-	-	0.99 mg/dL	40.00 U/L	42.00 U/L	25.00 U/L	20.00 mg/L	6.26 x10^3/μL	2.90 x10^3/μL	2.62 x10^3/μL	0.54 x10^3/μL
21	-	-	1.06 mg/dL	-	-	-	14.70 mg/L	6.77 x10^3/μL	3.37 x10^3/μL	2.60 x10^3/μL	0.58 x10^3/μL
22	1.07 mg/L	3.31 μg/L	1.07 mg/dL	-	-	-	10.1 mg/L 0	7.02 x10^3/μL	4.10 x10^3/μL	2.12 x10^3/μL	0.57 x10^3/μL
23	-	-	1.09 mg/dL	-	-	-	7.80 mg/L	6.00 x10^3/μL	4.58 x10^3/μL	1.30 x10^3/μL	0.12 x10^3/μL
38	2.49 mg/L	2.40 μg/L	1.07 mg/dL	203.00 U/L	49.00 U/L	67.00 U/L	7.80 mg/L	10.66 x10^3/μL	9.10 x10^3/μL	1.25 x10^3/μL	0.25 x10^3/μL
51	4.13 mg/L	1.98 μg/L	1.12 mg/dL	41.00 U/L	110.00 U/L	41.00 U/L	0.50 mg/L	10.26 x10^3/μL	7.39 x10^3/μL	2.27 x10^3/μL	0.46 x10^3/μL
123	3.35 mg/L	2.31 μg/L	1.07 mg/dL	22.00 U/L	18.00 U/L	10.00 U/L	5.00 mg/L	6.16 x10^3/μL	3.66 x10^3/μL	1.89 x10^3/μL	0.54 x10^3/μL

Table S1. History of measurements of plasma concentrations of mycophenolate (performed by LC-MS/MS method), everolimus (performed by immunoassay method), kidney function values, liver function values, C-reactive protein values, and leukocyte population counts. MP: mycophenolate; sCr: serum creatinine; AST/GOT: aspartate aminotransferase/glutamate oxaloacetate transaminase; ALT/GPT: alanine aminotransferase/glutamate pyruvate transaminase; GGT: gamma-glutamyl transferase; CRP: C-reactive protein; WBC: white blood cells.
